# The Production of Testosterone and Gene Expression in Neonatal Testes of Rats Exposed to Diisoheptyl Phthalate During Pregnancy is Inhibited

**DOI:** 10.3389/fphar.2021.568311

**Published:** 2021-04-12

**Authors:** Bin Ji, Zina Wen, Chaobo Ni, Qiqi Zhu, Yiyan Wang, Xiaoheng Li, Ying Zhong, Ren-Shan Ge

**Affiliations:** ^1^ Department of Anesthesiology and Perioperative Medicine, The Second Affiliated Hospital and Yuying Children’s Hospital of Wenzhou Medical University, Key Laboratory of Anesthesiology of Zhejiang Province, Wenzhou Medical University, Wenzhou, China; ^2^ Department of Obstetrics and Gynecology, The Second Affiliated Hospital and Yuying Children’s Hospital of Wenzhou Medical University, Wenzhou, China; ^3^ Chengdu Jinjiang Maternal and Child Health Hospital and Chengdu Xi’nan Gynecology Hospital, Chengdu, China

**Keywords:** diisoheptyl phthalate, fetal leydig cell, testis dysgenesis, leydig cell cluster, multinucleated gonocytes

## Abstract

**Background:** Diisoheptyl phthalate (DIHP) is a phthalate plasticizer, which is a branched phthalate. Here, we reported the effects of gestational exposure to DIHP on testis development in male rats.

**Methods:** Pregnant Sprague-Dawley rats were orally fed with vehicle (corn oil, control) or DIHP (10, 100, 500, and 1,000 mg/kg) from gestational day (GD) 12–21. At GD21, serum testosterone levels, the number and distribution of fetal Leydig cells, and testicular mRNA and protein levels, the incidence of multinucleated gonocytes, and focal testicular hypoplasia in the neonatal testis were measured.

**Results:** DIHP increased the fetal Leydig cell cluster size and decreased the fetal Leydig cell size with LOAEL of 10 mg/kg. DIHP did not affect the fetal Leydig cell number. DIHP significantly lowered serum testosterone levels, down-regulated the expression of steroidogenesis-related genes (*Lhcgr*, *Star*, *Cyp11a1*, *Hsd3b1*, *Cyp17a1*, and *Hsd17b3*) and testis descent-related gene (*Insl3*) as well as protein levels of cholesterol side-chain cleavage enzyme (CYP11A1) and insulin-like 3 (INSL3). DIHP dose-dependently increased the percentage of multinucleated gonocytes with the low observed adverse-effect level (LOAEL) of 100 mg/kg. DIHP induced focal testicular hypoplasia.

**Conclusion:** Gestational exposure to DIHP causes testis dysgenesis in rats.

## Introduction

Diisoheptyl phthalate (DIHP, CAS number 71888–89–6) is a synthetic phthalate plasticizer. It is an ester formed by one molecule of phthalic acid and two molecules of branched carbon chain alcohol. It belongs to a branched phthalate ester with six carbon atoms in the carbon backbone (Supplementary Figure S1). DIHP is used for vinyl flooring, tiles, carpet backing, molding, and coating plastisol, and partially replacement for low molecular weight plasticizers (ECHA, 2011). In Australia, DIHP is used as a PVC plasticizer and as screen printing ink. DIHP may exist in lubricating oil (ECHA, 2011). In the United States, the annual consumption of phthalates is estimated to exceed 470 million pounds (EPA, 2012), and DIHP is one of them. DIHP can be leached from plastics containing DIHP into environments such as food and water. Although there are no data on the absorption, distribution, metabolism, and excretion of DIHP in animal models and humans (ECHA, 2011), like other phthalates in the human body, DIHP may be rapidly converted to mono-ortho phthalate metabolites by lipase (Holm et al., 2004).

Many phthalates have been shown to have developmental and reproductive toxicity, and play the most significant role in inducing TDS in a rat model (Mao et al., 2019). The term TDS is used to describe the abnormal development of the reproductive tract of male neonates, including cryptorchidism and hypospadias, male infertility and testicular cancer in adult males induced by unexplained fetal causes (Skakkebaek et al., 2001). Total dysfunction of fetal Leydig cells, Sertoli cells, and germ cells after exposure to phthalates may cause TDS (Hu et al., 2009). One of these results is the inhibition of testosterone synthesis in fetal Leydig cells, which can be measured by the reduction of anogenital distance (AGD) in rodents and humans, which is a biomarker of androgen deficiency (Wolf et al., 1999; Hoshino et al., 2005; Swan et al., 2005; Borch et al., 2006).

Human epidemiological studies have shown that the boy’s AGD was inversely related to the concentration of phthalate metabolites in urine (Swan et al., 2005). In the study of rats, DIHP can reduce AGD and testicular testosterone levels (Hannas et al., 2011). Since testosterone synthesis mainly occurs in fetal Leydig cells, many genes in the testosterone synthesis cascade may be sensitive to the regulation of DIHP. However, the role of DIHP in down-regulating the gene expression of fetal Leydig cells is still unknown. The testosterone synthesis cascade includes luteinizing hormone receptor (LHCGR) signaling, scavenger receptor class B member 1 (SR-BI) after binding to high-density lipoprotein for cholesterol uptake, steroidogenic acute regulatory protein (StAR) for cholesterol transport to mitochondrion, as well as a set of testosterone synthases, including cholesterol side-chain cleavage enzyme (CYP11A1), 3β-hydroxysteroid dehydrogenase isoform 1 (HSD3B1), 17α-hydroxylase/17,20-lyase (CYP17A1), and 17β-hydroxysteroid dehydrogenase isoform 3 (HSD17B3) (Chen et al., 2020). Lower serum and testicular testosterone levels are one of the typical manifestations of phthalate-mediated TDS (Fisher et al., 2003; Hutchison et al., 2008; Hu et al., 2009). Other phthalate-mediated manifestations of TDS in rodents include abnormal aggregation of fetal Leydig cells and an abnormal increase in multinucleated gonocytes (MNGs) [see review (Hu et al., 2009)]. Cryptorchidism is usually induced by phthalates in rodent models and may be caused by reduction of testosterone and insulin-like 3 (INSL3) synthesis by fetal Leydig cells. INSL3 is a key peptide for the initial decline of fetal testes (Zimmermann et al., 1999; Adham et al., 2000; Emmen et al., 2000). In the current study, we report that DIHP affects these parameters of TDS and testicular gene expression.

## Materials and Methods

### Materials and Animals

Chemicals, reagents, kits, equipment, and software are listed in Supplementary Table S1. The primers for the analyzed genes are listed in Supplementary Table S2. 90 days-old male and female Sprague-Dawley rats were obtained from Shanghai Experimental Animal Center (Shanghai, China). All animal studies were conducted in accordance with the research protocol approved by the Animal Protection and Use Committee of Wenzhou Medical University, and the guidelines for the care and use of laboratory animals was followed.

### Administration of Diisoheptyl Phthalate to Animals

After acclimation for 7 days at the Animal Center of Wenzhou Medical University, the rats were mated. Thirty dams were randomly divided into five groups: 0, 10, 100, 500, or 1,000 mg/kg body weight DIHP, six in each group. Rats in the 0 mg/kg DIHP group (as the control group) received the same volume of corn oil. The dam was housed in an individually ventilated cage. The conditions of the animal room are set as follows: temperature 21–25°C, humidity 50–60%, light-dark cycle for 12 h. DIHP was suspended in corn oil for gavage. From the 12th to the 21st day of gestation (GD), the female rats were gavaged with DIHP with 0, 10, 100, 500 or 1,000 mg/kg bodyweight every day (Supplementary Figure S1). Based on previous study (Li et al., 2014), the duration of gavage is within the fetal Leydig cell development window. The dose of DINP was based on previous observation (Hannas et al., 2011), that is, DIHP can reduce testicular testosterone levels at 900 mg/kg. In our previous study (Hu et al., 2018), we also selected a similar dose range and regimen based on the most widely used phthalate (diethylhexyl phthalate, DEHP), using 10, 100, 500, and 1,000 mg/kg/day. An aliquot of 0.5 ml was taken for each gavage. Rats were delivered on GD21. The dam and male pups were euthanized by carbon dioxide on GD21. The weight of the dam was recorded before and during the treatment. The birth rate of the dam, the number of pups per dam, and the male to female ratio were calculated. The bodyweight and AGD of each male pup were collected. The testes of each male pup were weighed and used in the following experiment.

### Serum Testosterone Analysis

Serum testosterone determination was performed as described previously (Lin et al., 2008). The testosterone concentration was measured by radioimmunoassay as described (Akingbemi et al., 2001). The coefficient of variation between batches and within batches was within 15%.

### Enzymatic Staining of HSD3B1 to Identify Fetal Leydig Cells.

HSD3B1 is a biomarker for fetal Leydig cells, and was used to identify fetal Leydig cells. As mentioned previously (Lin et al., 2008), HSD3B1 was enzymatically stained in frozen sections of newborn testes. In brief, each group of frozen testes was embedded in a tissue-array module. The array block was cut to 10-micron sections in the cryostat. Sections were taken and attached to glass slides every five selections. The sections were stained with a staining solution containing 0.4 mM etiocholanolone, 2 mM NAD^+^ and tetranitroblue tetrazolium in phosphate buffered saline (PBS, pH 7.2). The sections were incubated in a humidified chamber at 37°C for 30 min. As a negative control, another section was incubated without etiocholanolone. The sections were washed with PBS and fixed with 4% PBS-buffered paraformaldehyde. Sections were counterstained with DAPI. An excitation wavelength of 350 nm was selected and combined with a bright field fluorescence microscope for photography.

### Count of Fetal Leydig Cells

As described previously (Lin et al., 2008), each of the above testis slices was selected and fetal Leydig cells were counted. The number of fetal Leydig cells per testis was calculated by stereology as previously described (Lin et al., 2008).

### Calculation of Fetal Leydig Cell Cluster Frequency

Each of the above testis slices was selected and stained with HSD3B1 enzyme solution to count single fetal Leydig cells or cluster containing two or more fetal Leydig cells and the frequency of fetal Leydig cell clusters was calculated as previously described (Lin et al., 2008). The size of the fetal Leydig cell cluster is defined as a single cell (1 fetal Leydig cell per cluster), small (2–4 fetal Leydig cells per cluster), medium (5–16 fetal Leydig cells per cluster) and large (greater than 16 fetal Leydig cells per cluster) (Li et al., 2014).

### Immunohistochemistry

The immunohistochemical method of testicular protein was as described previously (Guo et al., 2013). Briefly, after the Bouin’s solution was fixed, the neonatal testes were embedded in paraffin, prepared in the form of a tissue-array, and cut into 6-micron thick cross-sections under a microtome. Sections were stained using the staining kit described previously (Guo et al., 2013). In brief, the endogenous peroxidase of sections was blocked by H_2_O_2_, antigens were displayed by heating, primary antibodies (CYP11A1 and INSL3) were incubated, and then secondary antibodies were linked, and staining solution was added for the color display.

### Computer-Aided Image Analysis

CYP11A1 is a biomarker for fetal Leydig cells. As previously described (Guo et al., 2013), eight fields of views were randomly selected in each of three nonadjacent sections per testis and photos were taken. Image-Pro Plus analysis software was used to display fetal Leydig cells, the cell size and nuclear size of each fetal Leydig cell were analyzed, and the average area parameter was calculated. More than fifty fetal Leydig cells in each testis was measured, and the cell size and nuclear size and their ratio were averaged.

### Evaluation of Local Testicular Hypoplasia

Desmin is a biomarker of peritubular myoid cells. The seminiferous tubule is circled by peritubular myoid cells in cross-section, representing the normal testicular structure. As described previously (Li et al., 2014), ruptured seminiferous tubules represent focal testicular hypoplasia. Immunohistochemical detection of desmin using an anti-desmin antibody was performed. The testis with focal testicular hypoplasia was counted. A testis section containing at least one focal testicular hypoplasia is designated as focal testicular hypoplasia testis.

### Semi-quantitative Immunohistochemical Detection of Protein Levels

CYP11A1 is an important rate-limiting testosterone synthase in fetal Leydig cells. INSL3 is an important peptide secreted by fetal Leydig cells and can induce testis descent (Adham et al., 2000). Immunohistochemical staining using antibodies CYP11A1 and INSL3 was performed. Image-Pro Plus analysis software was used to measure the density of the cytoplasmic portion of CYP11A1 or INSL3 and background area and the average density parameter was calculated as described previously (Li et al., 2014). More than fifty fetal Leydig cells were evaluated in each testis and the net density of each sample was averaged and analyzed.

### Evaluation of Multinucleated Gonocytes

As described previously (Li et al., 2014), hematoxylin and eosin (HE) were used to stain testis sections. An image of seminiferous cords was taken. A complete testis cross-section of each animal was analyzed, and the percentage of seminiferous cord containing at least one MNG was recorded. The percentage of cords containing MNG in the total cords counted was analyzed.

### Real-Time Quantitative PCR

As mentioned previously (Li et al., 2014), total RNA was extracted from rat testis using TRIzol kit. cDNA synthesis and subsequent qPCR were performed as previously described (Lin et al., 2008). The ribosomal protein S16 (*Rps16*) mRNA level in each sample was determined as an internal control. The genes measured include fetal Leydig cell genes: luteinizing hormone receptor (*Lhcgr*); scavenger receptor class B member 1 (*Scarb1*), which encodes cholesterol high-density lipoprotein receptor; steroidogenic acute regulatory protein (*Star*), which encodes a cholesterol transporter StAR; a group of testosterone synthases CYP11A1 (*Cyp11a1*), HSD3B1 (*Hsd3b1*), CYP17A1 (*Cyp17a1*), and HSD17B3 (*Hsd17b3*); INSL3 gene (*Insl3*) for testis descent. A Sertoli cell gene *Dhh*, which encodes desert hedgehog to regulate fetal Leydig cell development (Yao et al., 2002), was also measured. The relative mRNA levels of test genes were normalized to *Rps16* as previously described (Lin et al., 2008).

### Statistical Analysis

Values are expressed as mean ± SEM (*n* = 6dams), and the data were analyzed by repeated measured one-way ANOVA with Dunnett's multiple comparisons tests. The lowest observed adverse effect level (LOAEL) was determined. The half-maximum effective dose (EC_50_) and half-maximum inhibitory dose (IC_50_) values were evaluated by dose-response nonlinear regression by least-squares fit using GraphPad Prism version 6. Dose-response percent of control data for serum testosterone and mRNA levels were analyzed by nonlinear four-parameter regression analysis with the top constrained to 100% and the bottom to 0% of control using sigmoidal fit with the variable slope in the GraphPad Prism version 6 (GraphPad Software, Inc., La Jolla, CA, United States). Differences between dose-response curves were determined by comparing ED_50_ or IC_50_ values among the phthalate (*p* < 0.05) using GraphPad Prism software.

## Results

### Diisoheptyl Phthalate Shortens Anogenital Distance of Male Fetus

We evaluated general toxicological parameters after exposure to DIHP. As shown in Table 1, DIHP did not affect the bodyweight of the dam. DIHP did not affect the birth rate of the dam, the number of pups per dam, and the sex ratio of male to female pups. However, DIHP significantly reduced the birth weight of male pups (500 and 1,000 mg/kg). DIHP shortened AGD at doses of 500 and 1,000 mg/kg, respectively, (Table 1), indicating that DIHP blocks testosterone synthesis.

**TABLE 1:** Toxicological parameters before and after exposure to diisoheptyl phthalate (DIHP).

Parameters	DIHP, mg/kg/day (oral)				
	0	10	100	500	1,000
Dams					
Number of dams	6	6	6	6	6
Body weight at GD 0, g	248.8 ± 6.2	243.0 ± 7.2*	241.7 ± 7.6*	243.3 ± 4.0*	245.2 ± 5.0*
Body weight at GD 12, g	291.5 ± 1.71	294.2 ± 3.3*	293.8 ± 3.3*	295.3 ± 3.1*	290.2 ± 3.0*
Body weight at GD 21, g	407.5 ± 2.2	402.7 ± 3.4*	401.2 ± 5.2*	402.2 ± 3.5*	395.0 ± 3.0*
Pup numbers per dam	14 ± 2	14 ± 3*	15 ± 2*	12 ± 5*	11 ± 5*
Birth rate	6/6	6/6	6/6	6/6	6/6
Pup male (%)	48 ± 12	49 ± 15*	51 ± 11*	48 ± 9*	49 ± 14*
Male pups					
Body weight, g	7.4 ± 0.3	6.9 ± 0.54*	6.6 ± 0.4*	6.4 ± 0.3*	6.0 ± 0.2**
AGD, mm	3.6 ± 0.2	3.4 ± 0.2*	2.7 ± 0.3*	2.4 ± 0.2**	2.3 ± 0.3***

Dams of Sprague-Dawley rats were gavaged with DIHP from gestational day (GD) 12–20. Values are mean ± SEM.

*p > 0.05, **p < 0.05, ***p < 0.01, compared to control (DIHP, 0 mg/kg).

### Diisoheptyl Phthalate Induces Abnormal Fetal Leydig Cell Aggregation

**FIGURE 1:**
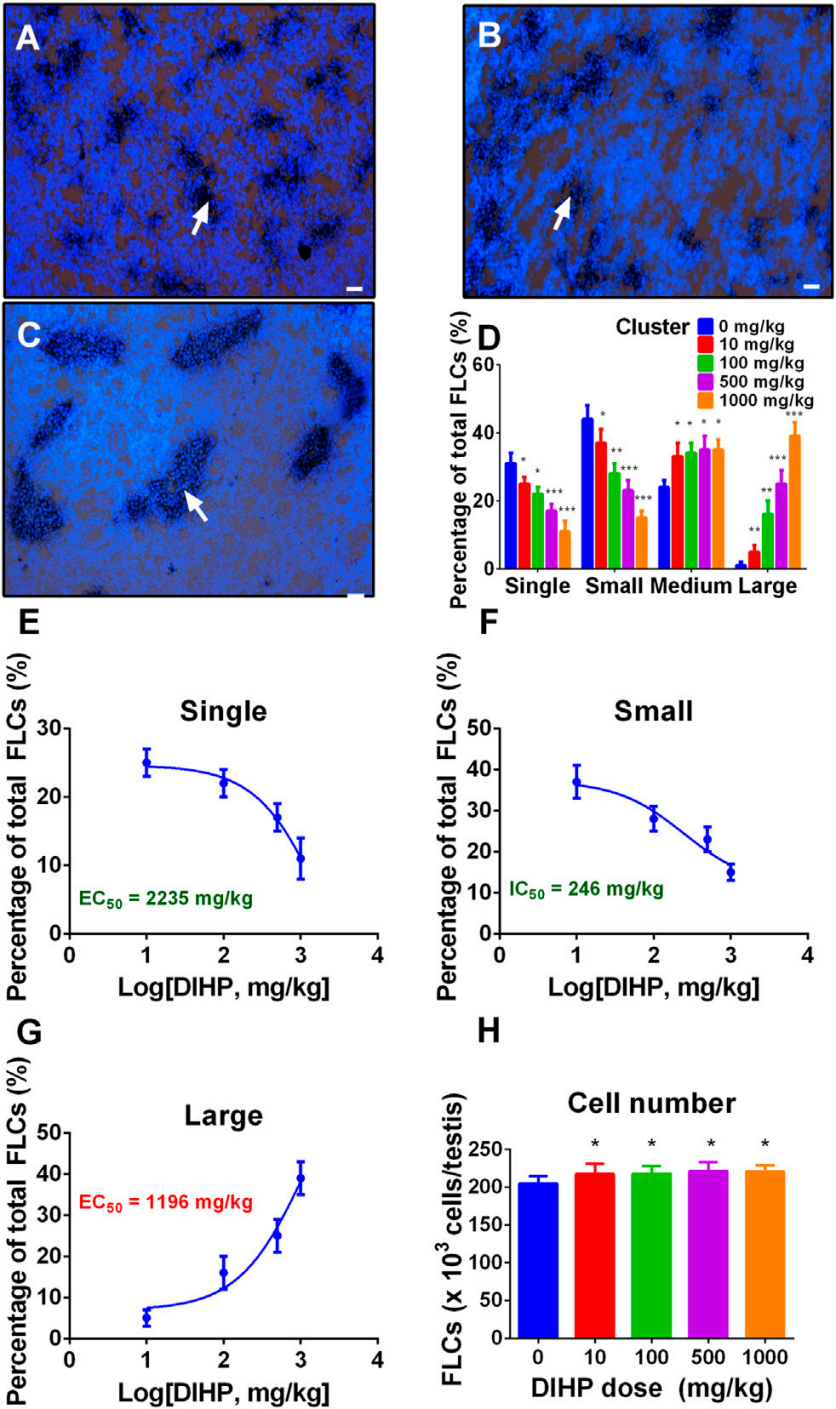
Effect of diisoheptyl phthalate (DIHP) on the distribution and cell number of fetal Leydig cells (FLCs). Representative micrographs of testicular slices of 0 **(A)**, 100 **(B)**, and 1,000 **(C)** mg/kg DIHP. Scale bar = 50 μm. White arrows point to a bunch of fetal Leydig cells. Group **(D)**, Fetal Leydig cell allocation; Panel **(E–G)**, IC_50_ or EC_50_ values are used for single, small, and large fetal Leydig cell clusters; Panel **(H)**, Fetal Leydig cell number; data expressed as mean ± SEM, *n* = 6; *p > 0.05, **p < 0.05, ***p < 0.01 indicate that the significant level between the DIHP group and the control group, respectively.

Fetal Leydig cells can be either a single cell or a cluster of two or more cells (Wen et al., 2016). We designated the fetal Leydig cell clusters as single (single cell per cluster), small (2-4 cells per cluster), medium (5–16 cells per cluster), and large cluster (more than 16 cells per cluster). As shown in Figure 1, DIHP significantly increased the large clusters of fetal Leydig cells (Figure 1D). DIHP significantly reduced the single-cell population (Figure 1E) and the small cluster populations (Figure 1F). DIHP reduced the single cell population with a LOAEL of 500 mg/kg (Figure 1D) and an IC_50_ value of 2,235 mg/kg (Figure 1E). DIHP reduced the population of small cluster, with a LOAEL of 100 mg/kg (Figure 1D) and an IC_50_ value of 246 mg/kg (Figure 1F). In contrast, DIHP increased the large cluster population with a LOAEL of 10 mg/kg (Figure 1D) and an EC_50_ value of 1,196 mg/kg (Figure 1G). DIHP did not affect the medium cluster-sized population (Figure 1D). Fetal Leydig cell clusters can reach more than 100 fetal Leydig cells per cluster at a concentration of 1,000 mg/kg. These data indicate that fetal Leydig cells tend to aggregate abnormally after exposure to DIHP.

### Diisoheptyl Phthalate Does not increase the Number of Fetal Leydig Cells

We counted the fetal Leydig cells of each testis and found that DIHP did not affect the number of fetal Leydig cells at any dose (Figure 1H). This indicates that the increase in the fetal Leydig cell cluster size is not due to the increase in the number of fetal Leydig cells.

### Diisoheptyl Phthalate Lowers Fetal Leydig Cell Size

**FIGURE 2:**
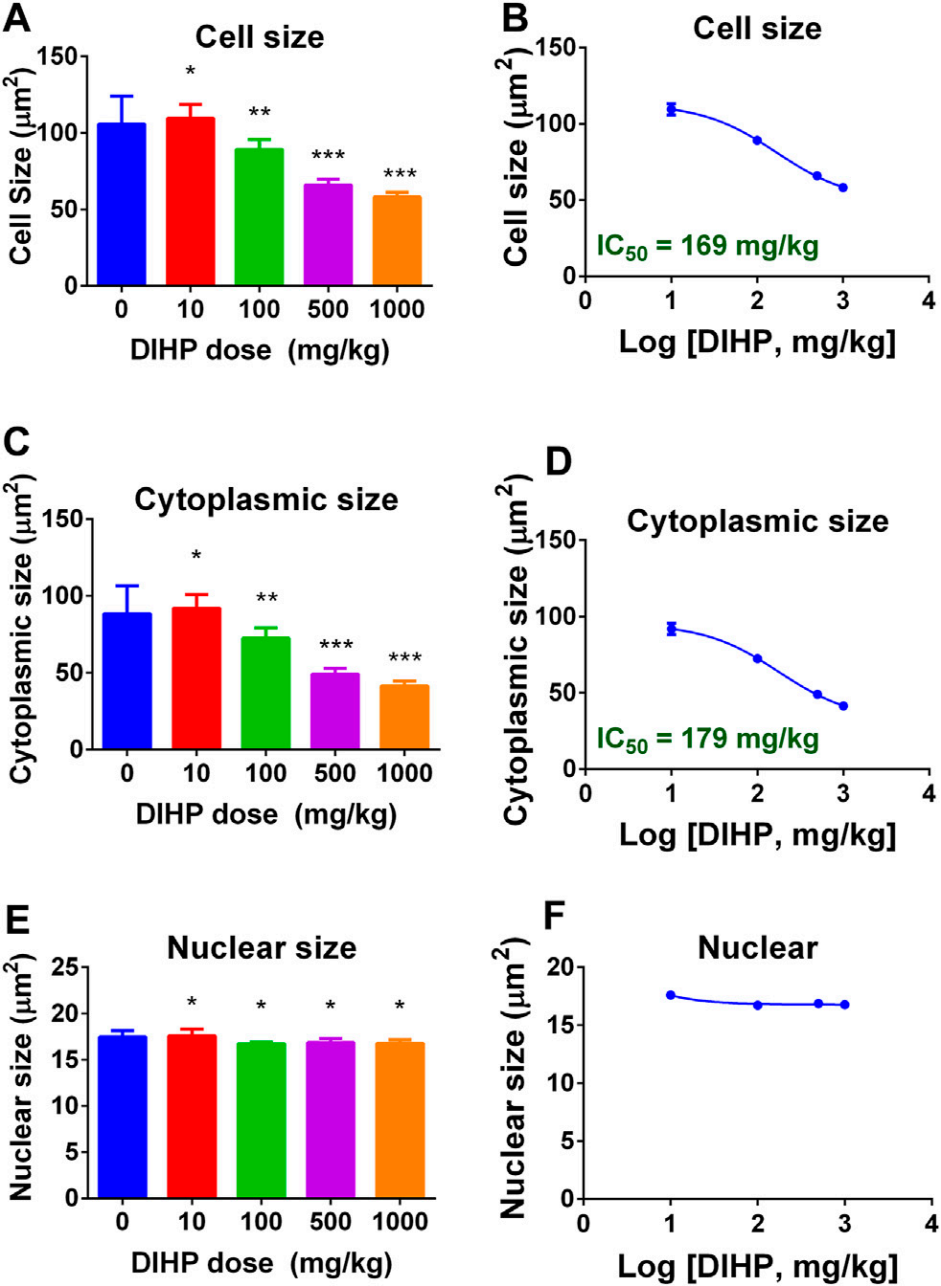
Effect of DIHP exposure on fetal Leydig cell (FLC) cell, cytoplasm, and nuclear size. From GD12 to GD21, the dams were gavaged with 0 (control), 10, 100, 500, and 1,000 mg/kg DIHP. The data are expressed as mean ± SEM. Panels **(A, B)**: cell size; Panels **(C, D)**: cytoplasm size; Groups **(E, F)**: nuclear size; Groups B, D, and F: IC_50_ values. Data are expressed as mean ± SEM, *n* = 6; *p > 0.05, **p < 0.05, ***p < 0.01 indicate that the significant level between the DIHP group and the control group, respectively.

DIHP dose-dependently reduced fetal Leydig cell size (Figure 2A) and cytoplasm size (Figure 2C) with a LOAEL of 100 mg/kg without affecting nuclear size (Figures 2E,F). IC_50_ values for lowering cell size (Figure 2B) and cytoplasm size (Figure 2D) were 169 and 179 mg/kg, respectively. This indicates that DIHP blocks the development of fetal Leydig cells.

### Diisoheptyl Phthalate Decreases Serum Testosterone Level of Male Pups

**FIGURE 3:**
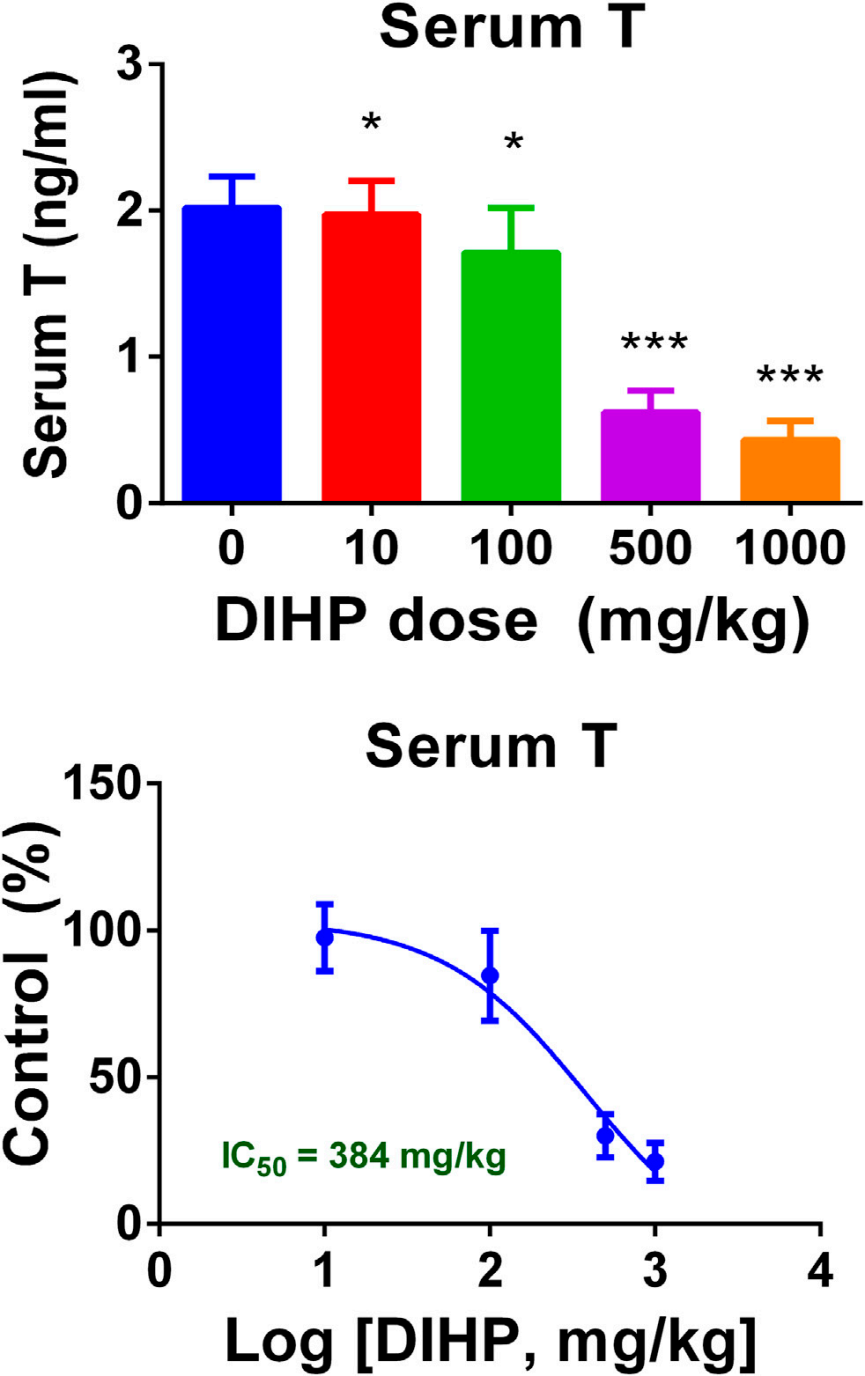
Effect of DIHP exposure during pregnancy on serum testosterone (T) levels. From GD12 to GD21, the dams were gavaged with 0 (control), 10, 100, 500, and 1,000 mg/kg DIHP. **(A)** Serum T level; **(B)** IC_50_ value of DIHP; data expressed as mean ± SEM, *n* = 6; *p > 0.05, ***p < 0.01 indicate that the significant level between the DIHP group and the control group, respectively.

As shown in Figure 3, DIHP dose-dependently decreased serum testosterone levels with a LOAEL of 100 mg/kg (Figure 3A) and an IC_50_ value of 384 mg/kg (Figure 3B). This indicates that DIHP disrupts testosterone synthesis of fetal Leydig cells.

### Diisoheptyl Phthalate Down-regulates Testicular Cell Gene Expression

**FIGURE 4:**
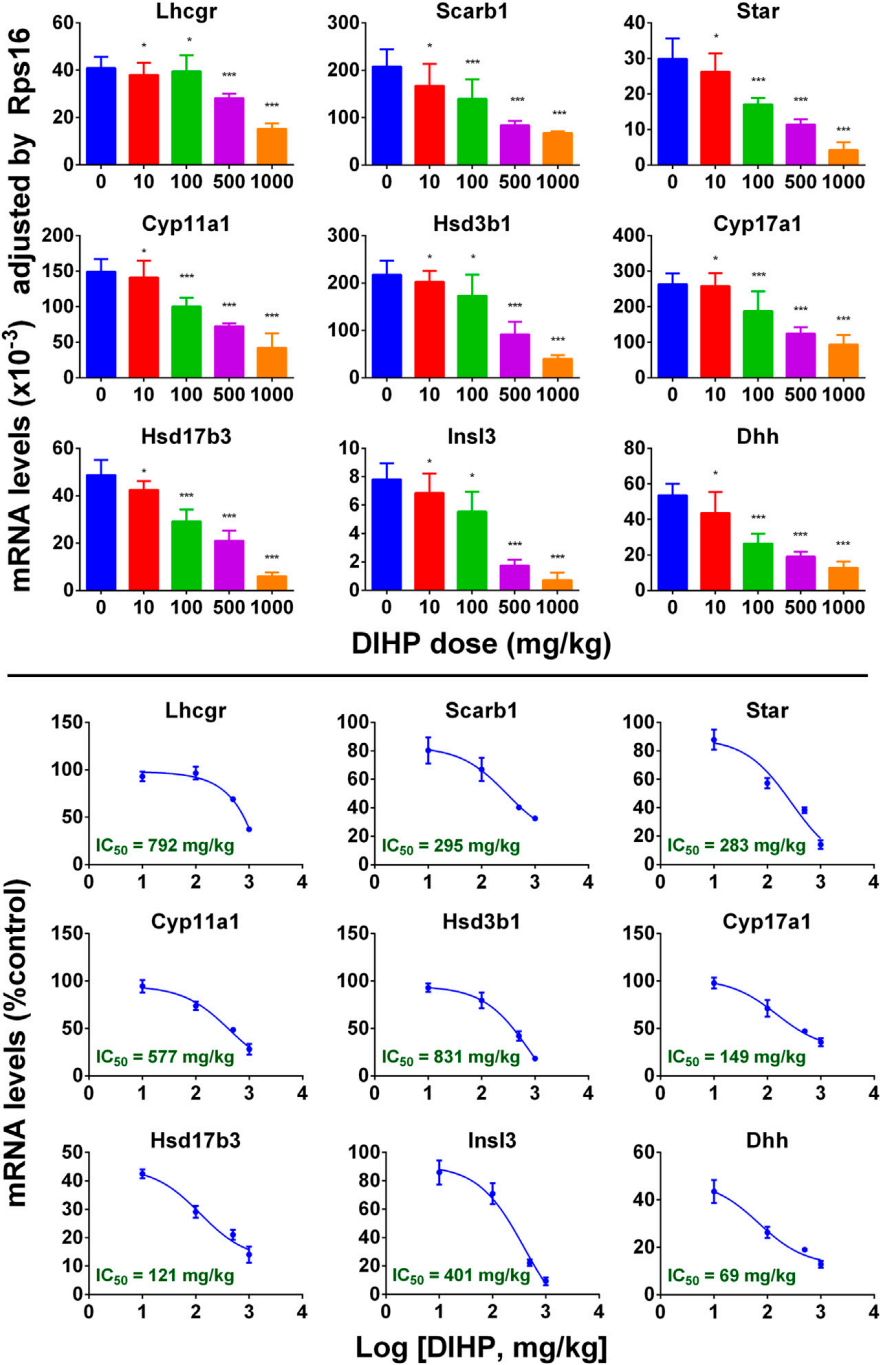
qPCR analysis of mRNA levels in testes after exposure to DIHP in pregnancy. The dam was gavaged with different doses of DIHP from GD12 to GD21. Lower panel: IC_50_ value. Data are expressed as mean ± SEM, *n* = 6; *p > 0.05, **p < 0.05, ***p < 0.01 indicate that the significant level between the DIHP group and the control group, respectively.

A group of mRNAs specific for fetal Leydig cells and Sertoli cells were selected to investigate the effect of DIHP. The genes are: *Lhcgr*, *Scarb1*, *Star*, *Cyp11a1*, *Cyp17a1*, *Hsd3b1*, *Hsd17b3*, *Insl3,* and *Dhh*. DIHP dose-dependently down-regulated *Lhcgr*, *Scarb1*, *Star*, *Cyp11a1*, *Cyp17a1*, *Hsd3b1*, *Hsd17b3*, *Insl3,* and *Dhh*. The IC_50_ values of *Lhcgr*, *Scarb1*, *Star*, *Cyp11a1*, *Cyp17a1*, *Hsd3b1*, *Hsd17b3*, *Insl3,* and *Dhh* were 792, 295, 283, 577, 831, 149, 121, 401, and 69 mg/kg (Figure 4). These indicate that DIHP has different efficacy to affect the expression of fetal testis genes.

### Diisoheptyl Phthalate Reduces Protein Levels of CYP11A1 and INSL3

**FIGURE 5:**
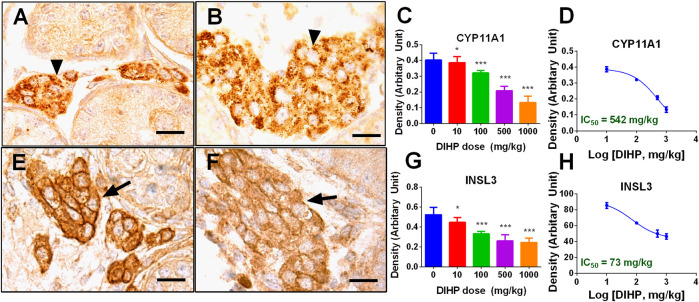
Semi-quantitative immunohistochemical analysis of CYP11A1 and INSL3 protein levels after exposure to DIHP in pregnancy. Figure **(A–D)**: CYP11A1; Panel **(E–H)**: DIHP. Representative images **(A–B)**: CYP11A1 stained at 0 and 1,000 mg/kg DIHP; **(E–F)**: INSL3 stained at 0 and 1,000 mg/kg DIHP; Panels **(D, H)**: IC_50_ values. Mean ± SEM, *n* = 6; *p > 0.05, ***p < 0.01 indicate that the significant level between the DIHP group and the control group, respectively.

Immunohistochemical staining of CYP11A1 and INSL3 was performed (Figure 5). Semi-quantitative analysis showed that DIHP can reduce the density of CYP11A1 and INSL3 proteins.

### Diisoheptyl Phthalate Increases Multinucleated Gonocyte

**FIGURE 6:**
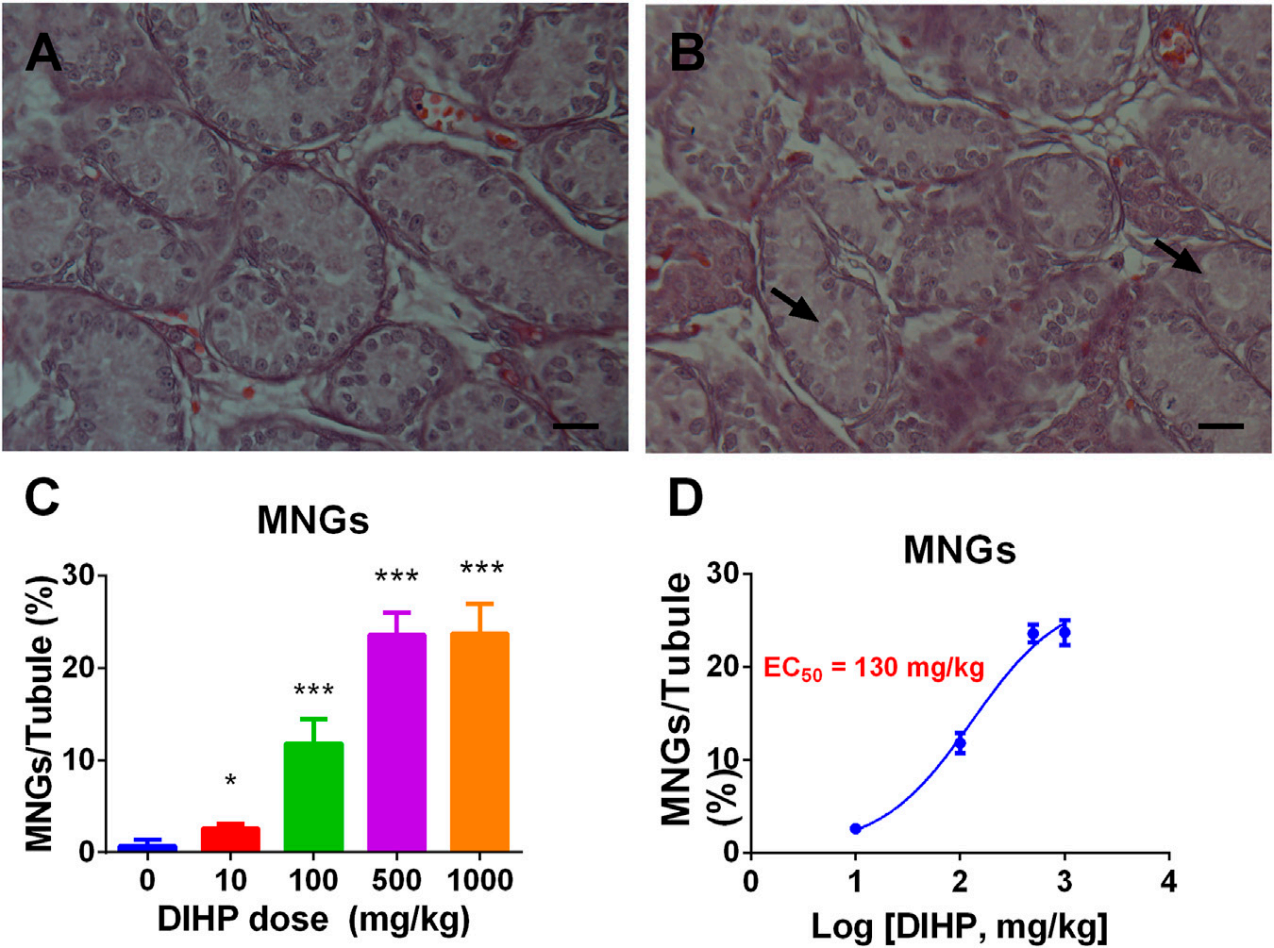
Effect of DIHP on the formation of multinucleated gonocyte (MNG). From GD 12 to GD21, 0 (control), 10, 100, 500, and 1,000 mg/kg DIHP were administered by gavage to dams. Measurements were made on GD 21. **(A, B)**: The arrow of the HE stained image of MNG at 0 and DIHP 1000 mg/kg group indicates the MNG in the DIHP-treated testis. **(C)** Quantitative results; **(D)** IC_50_ value. The data are expressed as mean ± SEM, *n* = 6; *p > 0.05, ***p < 0.01 indicate that the significant level between the DIHP group and the control group, respectively.

Many phthalates increase incidence of MNG (Mahood et al., 2005; Lin et al., 2008; Li et al., 2014). We examined the effects of DIHP on the formation of MNG. Testicular micrographs showed that MNG was barely detectable in the control testis (Figure 6A), and MNG was visible in the DIHP-treated testes (Figure 6B). DIHP dose-dependently increased the incidence of MNG (Figure 6C), LOAEL was 100 mg/kg (Figure 6C), and EC_50_ value was 130 mg/kg (Figure 6D). This indicates that DIHP induces MNG.

### Diisoheptyl Phthalate Increases the Incidence of Focal Testicular Hypoplasia

**FIGURE 7:**
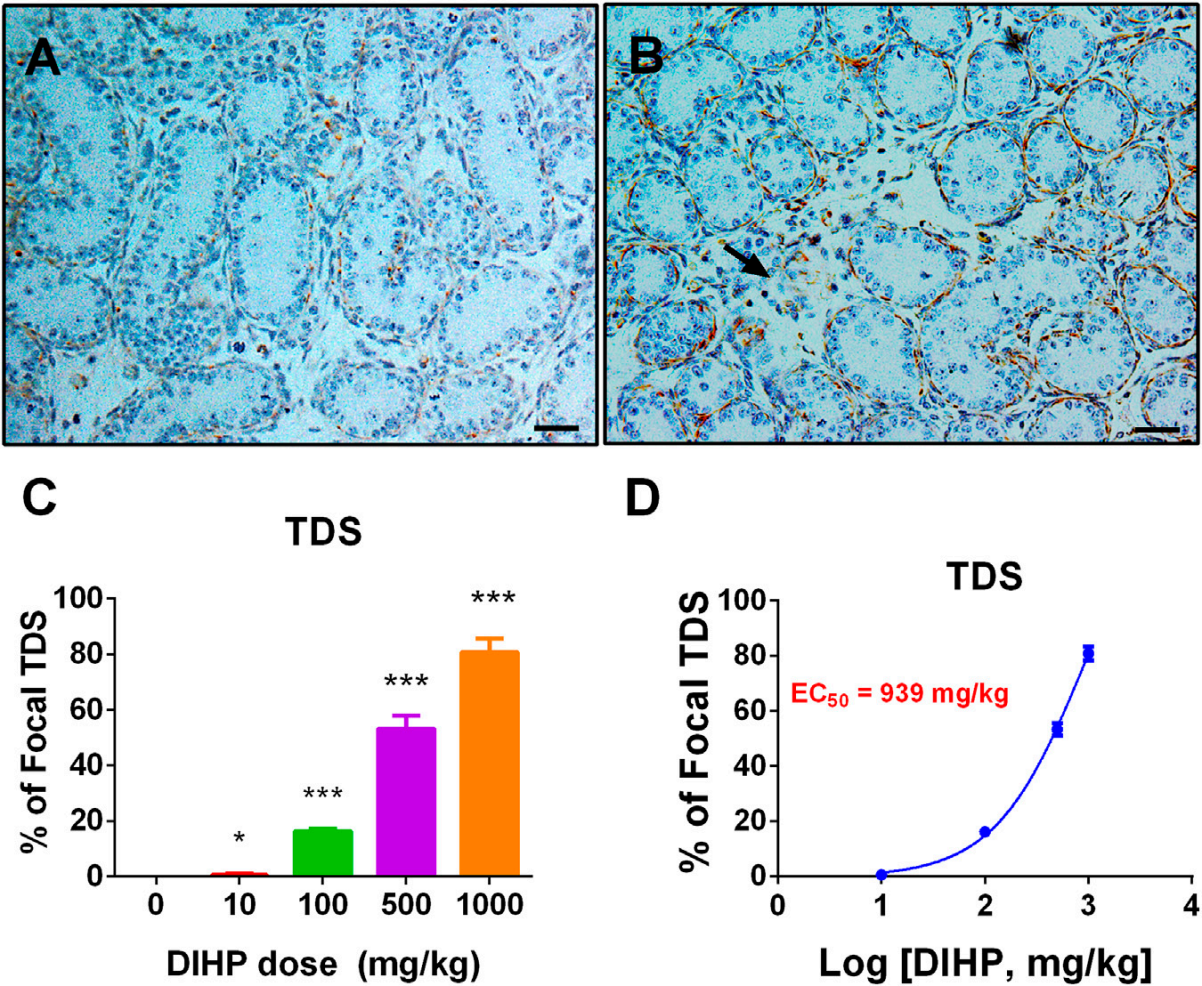
Effect of DIHP exposure on focal testicular hypoplasia. From GD 12 to GD21, 0 (control), 10, 100, 500, and 1,000 mg/kg DIHP were administered by gavage to dams. Panels **(A, B)**: Immunohistochemical staining of DIHP at 0 and 1,000 mg/kg. Arrows indicate areas of focal hypoplasia; Scale bar = 50 microns; **(C)** quantitative results; **(D)** IC_50_ value. Data are expressed as mean ± SEM, *n* = 6; *p > 0.05, ***p < 0.01 indicate that the significant level between the DIHP group and the control group, respectively.

Some phthalates can induce focal testicular hypoplasia (Mahood et al., 2005; Mahood et al., 2006; Li et al., 2014). Desmin was used to show the integrity of seminiferous cords. In the control testes, there were no areas of focal testicular hypoplasia (Figure 7A). However, the incidence of focal testicular hypoplasia in the testes treated with DIHP increased (Figure 7B). Of the six testes in the 500 mg/kg DIHP group, three testes had at least one focal hypoplasia area (Figure 7C), and five testes in 1,000 mg/kg DIHP had focal hypoplasia. The EC_50_ value of DIHP-induced focal testicular hypoplasia is 939 mg/kg. This indicates that DIHP causes focal testicular hypoplasia.

## Discussion

In the current study, pregnant Sprague-Dawley rat was gavaged with 0, 10, 100, 500, and 1,000 mg/kg DIHP from GD 12 to 21 every day. With the increase of MNGs and focal testicular hypoplasia, the decrease of AGD, and the reduction of serum testosterone level, DIHP obviously induced TDS. Further research has shown that DIHP induced abnormal fetal Leydig cell aggregation and down-regulated the expression of genes related to steroid production (*Lhcgr, Star, Cyp11a1, Hsd3b1, Cyp17a1,* and *Hsd17b3*) and earlier testis descent (*Insl3*) and growth factor secreted by Sertoli cells (*Dhh*).

After gestational exposure, DIHP caused abnormal aggregation of fetal Leydig cells with a LOAEL of 10 mg/kg (Figure 1). Previous studies have shown that exposure to other phthalates (including DBP, DCHP, DEHP, and DINP) during pregnancy can cause abnormal aggregation of fetal Leydig cells (Mahood et al., 2005; Mahood et al., 2007; Lin et al., 2008; Li et al., 2014; Li et al., 2016). The LOAEL of fetal Leydig cell aggregation induced by DBP (Chen et al., 2017), DCHP (Li et al., 2016), DEHP (Lin et al., 2008), and DINP (Li et al., 2014) was 10 mg/kg. This indicates that DIHP is comparable to the above-mentioned phthalates in inducing the aggregation of fetal Leydig cells. Although there is evidence that abnormal aggregation of fetal Leydig cells was associated with male infertility (Mahood et al., 2006) and cryptorchidism (Wilson et al., 2004), its physiological consequences are still unknown. After exposure to DIHP during pregnancy, the mechanism of abnormal fetal Leydig cell aggregation is unclear. One possible explanation is that fetal Leydig cell aggregation is induced by growth factors or cytokines secreted by testicular cells. In a previous study, Lin et al. showed that after exposure to DEHP during pregnancy, locally produced leukemia inhibitory factor was significantly induced to cause the aggregation of fetal Leydig cells (Lin et al., 2008).

Further analysis showed that since the number of fetal Leydig cells remained unchanged, the formation of fetal Leydig cell aggregation in response to DIHP was not caused by an increase in the number of fetal Leydig cells (Figure 1H). Interestingly, exposure to DBP (Mahood et al., 2005; Mahood et al., 2007; Chen et al., 2017), DCHP (Li et al., 2016), and DINP (Li et al., 2014) did not change the fetal Leydig cell number, while DEHP decreased fetal Leydig cell number at 500 and 1,000 mg/kg (Lin et al., 2008). The effect of DIHP on fetal Leydig cell number is identical to that observed for DBP, DCHP, and DINP.

Previous studies have shown that gestational exposure to DBP (Foster et al., 2000; Mahood et al., 2005), BBP (Gray et al., 2000; Howdeshell et al., 2008), DEHP (Lin et al., 2008), DINP (Li et al., 2014), and DIHP (Hannas et al., 2011) may induce a range of reproductive/developmental toxicity, including cryptorchidism, hypospadias, and abnormal testicular development. This may be due to phthalate-induced inhibition of testosterone synthesis. Previous studies have shown that exposure of DIHP and DEHP from GD12 to GD18 can reduce testicular testosterone levels and have equivalent efficacy and that DEHP can down-regulate the expression of *Star* and *Cyp11a1* with IC_50_ values of 443 and 574 mg/kg (Hannas et al., 2011).

DIHP reduced the level of steroidogenesis-related transcripts. The IC_50_ values of DIHP for *Lhcgr*, *Scarb1*, *Star*, *Cyp11a1*, *Hsd3b1*, *Cyp17a1*, *Hsd17b3* were 792, 295, 841, 577, 831, 149, and 121 mg/kg/day (Figure 4). *Hsd17b3* and *Cyp17a1* are the most sensitive to DIHP. Previous studies have demonstrated that gestational exposure to DEHP from GD12 to GD18 in rats down-regulated *Star* and *Cyp11a1* with IC_50_ values of 443 and 574 mg/kg, respectively (Hannas et al., 2011). This indicates that DIHP has a similar potency range for down-regulating *Star* and *Cyp11a1* to DEHP.

Fetal Leydig cells secrete a peptide hormone (INSL3) that binds to the leucine-rich repeat-containing G protein coupled receptor eight in fetal gubernaculum (Zhang et al., 2009) and pulls testes down (Adham et al., 2000; Anand-Ivell et al., 2009). Lower expression of *Insl3* may cause cryptorchidism (Zimmermann et al., 1999). Several reports indicate that exposure to other phthalates (DEHP and DBP) during pregnancy can down-regulate expression of *Insl3* and induce cryptorchidism (Shono et al., 2000; Wilson et al., 2004; Mckinnell et al., 2005; Mahood et al., 2007). In the current study, we found that DIHP down-regulated *Insl3* expression with an IC_50_ value of 401 mg/kg. Since the IC_50_ value of *Insl3* was 589 mg/kg after exposure to DEHP. This indicates that DEHP is not as effective as DIHP in reducing *Insl3* expression.

DIHP down-regulated the expression of *Dhh* with an IC_50_ of 69 mg/kg/day (Figure 4). *Dhh* is a Sertoli cell gene, which encodes DHH. DHH is critical for fetal Leydig cell development. Knockout of *Dhh* in mice led to significant defects of fetal Leydig cell development (Yao et al., 2002). Therefore, the down-regulation of *Dhh* after exposure to DIHP may be the mechanism of DIHP-induced defect in the fetal Leydig cell development, including the lower expression of steroidogenesis-related genes and *Insl3,* as well as decreased fetal Leydig cell and cytoplasm size.

DIHP increased the occurrence of MNG. The EC_50_ value of DIHP was 130 mg/kg (Figure 6). The previous study has shown that an EC_50_ in the induction of MNG by DBP was at least 100 mg/kg (Chen et al., 2017). For phthalates, including DCHP, DEHP and DINP, there is also an increase in MNG (Lin et al., 2008; Li et al., 2014; Li et al., 2016).

DIHP increased the incidence of focal testicular hypoplasia. DIHP had an EC_50_ value of 939 mg/g (Figure 7). Phthalates, including DCHP, DEHP, and DINP, have also been reported to have focal testicular hypoplasia (Lin et al., 2008; Li et al., 2014; Li et al., 2016).

In the current study, we found that the developmental defects produced by DIHP in rat testis are very similar to the defects produced by other C4-C6 carbon skeleton phthalates, including BBP (Gray et al., 2000; Howdeshell et al., 2008), DBP (Foster et al., 2000; Mahood et al., 2005), DEHP (Lin et al., 2008), and DINP (Li et al., 2014). Indeed, DIHP caused a significant decrease in the serum testosterone level of male pups, with an IC_50_ value of 384 mg/kg/day (Figure 3B). After exposure to DIHP during pregnancy, this IC_50_ value for suppressing serum testosterone levels was comparable to that for testicular testosterone (IC_50_ = 410 mg/kg/day) (Hannas et al., 2011). DEHP has a similar potency to inhibit *ex vivo* testicular testosterone production *in vitro* (IC_50_ = 426 mg/kg/day) (Hannas et al., 2011).

DIHP is used as a plasticizer for polymers and is mainly used in floor manufacturing. Therefore, DIHP exposure may occur through the manufacturing process and floor use. As the temperature increases, the possibility of DIHP exposure during the floor manufacturing process increases. Consumers may also be exposed to DIHP when setting up new floors containing DIHP. DIHP exposure levels in dust (Wensing et al., 2005), fish (Mackintosh et al., 2004), oysters (Mackintosh et al., 2004), and mussels (Mackintosh et al., 2004) were comparable to butyl benzyl phthalate, one of the most abundant phthalates. There is no toxicokinetic data available for DIHP. However, the structure of DIHP is similar to another phthalate, di-n-hexyl phthalate (DNHP), which has a straight main chain. According to research of DNHP and other transitional phthalates, DIHP may be easily absorbed from the gut in the form of monoesters and excreted in the urine after being metabolized by the liver (CLH, 2012). The main metabolites of DIHP are monohydroxyheptyl phthalate, mono-oxoheptylphthalate, and monocarboxyhexyl phthalate. A recent survey of 205 subjects in the United States population from 2018 to 2019 showed that the geometric means of monocarboxyhexyl phthalate, monohydroxyheptyl phthalate, and mono-oxoheptylphthalate were 1.31, 0.59, and 0.03 ng/ml, respectively, and the sum of these three phthalate metabolites was about 2 ng/ml (Silva et al., 2019). When compared with the widely used DEHP, the average urine level of total DEHP metabolites in 378 United States pregnant women conducted in 2018 being about 14 ng/ml (Wenzel et al., 2018), the DIHP exposure was slightly lower. However, considering that phthalates have a cumulative effect on the development of fetal Leydig cells (Howdeshell et al., 2007; Howdeshell et al., 2008), the existence of DIHP should be concerned.

In conclusion, exposure to DIHP during pregnancy affects fetal Leydig cell number, distribution, and testosterone production capacity, induces MNG, and causes focal testicular hypoplasia. DIHP down-regulates the expression of many key genes in testosterone synthesis (*Lhcgr*, *Scarb1*, *Star*, *Cyp11a1*, *Hsd3b1*, *Cyp17a1*, and *Hsd17b3*), which leads to a decrease in the synthesis of testosterone, and the expression of *Insl3* that leads to the reduction of INSL3, resulting in developmental defects in the male reproductive tract, such as cryptorchidism and hypospadias. Our findings also provide valuable data that can be used to predict the risks associated with DIHP exposure in the uterus. Considering that its exposure level of DIHP is not as abundant as other commonly used phthalates (such as DBP), its effect on the male reproductive system may not be as effective as DBP.

## Data Availability

The original contributions presented in the study are included in the article/Supplementary Material, further inquiries can be directed to the corresponding authors.
