# Supplementary material for: The Production of Testosterone and Gene Expression in Neonatal Testes of Rats Exposed to Diisoheptyl Phthalate During Pregnancy is Inhibited
Source: Front Pharmacol. 2021 Apr 12;12:568311. doi: 10.3389/fphar.2021.568311 (PMC8072358; doi:10.3389/fphar.2021.568311)
Supplement: Supplementary file 1 [file table1.docx]

Supplementary Table S1. Chemicals, reagents, kits, equipment, and software

| **Name** | **Vendor (City, State)** |
| --- | --- |
| Antibody of CYP11A1 | Cell Signaling Technology (Danvers, MA) |
| Antibody of INSL3 | Abcam (San Francisco, CA) |
| Antibody of HSD3B1 | Abcam (San Francisco, CA) |
| Antibody of desmin | Abcam (San Francisco, CA) |
| BX53 microscope | Olympus (Tokyo, Japan) |
| Diisoheptyl phthalate | Sigma (St. Louis, MO) |
| Etiocholanolone | Sigma (St. Louis, MO) |
| GraphPad Prism 6 | GraphPad Software Inc. (San Diego, CA) |
| Hematoxylin and eosin | Sigma (St. Louis, MO) |
| Image-Pro 6 Plus software | Media Cybernetics (Silver Spring, MD) |
| NanoDrop (ND-2000) | Thermo-Fisher (Redwood City, CA) |
| NAD^+^ | Sigma (St. Louis, MO) |
| SYBR Green qPCR Kit | Takara (Otsu, Japan) |
| Tetranitroblue tetrazolium | Sigma (St. Louis, MO) |
| Trizol | Invitrogen (Carlsbad, CA) |
| Vector ABC Kit | Vector (Burlingame, CA) |
